# Precision medicine in renal stone-formers

**DOI:** 10.1007/s00240-018-1091-5

**Published:** 2018-11-20

**Authors:** Fay Hill, John A. Sayer

**Affiliations:** 1 Institute of Genetic Medicine, Newcastle University, Central Parkway, Newcastle, NE1 3BZ UK; 2 Renal Services, Newcastle upon Tyne NHS Foundation Trust, Freeman Road, Newcastle upon Tyne, NE7 7DN UK; 3 NIHR Newcastle Biomedical Research Centre, Newcastle upon Tyne, NE4 5PL UK

**Keywords:** Nephrolithiasis, Urolithiasis, Genetics, Precision medicine

## Abstract

Here we define precision medicine approaches and discuss how these may be applied to renal stone-formers to optimise diagnosis and a management. Using the gene discovery of monogenic stone disorders as examples, we discuss the benefits of personalising therapies for renal stone-formers to provide improved prevention and treatment of these disorders.

## Introduction

Renal stones, amongst the lay population, are infamous for their ability to cause severe agonising pain. With 1–5% of the world’s population estimated to develop nephrolithiasis at some point in their life [33], many people will recognise the cardinal symptoms of colic secondary to renal stone disease. The search to uncover genetic (usually within single genes) and genomic factors (within a person’s entire set of DNA sequence) predisposing to renal stone formation has been a more complex and often less commonly explored path [27, 28].

In order to discuss how a “precision medicine” (or sometimes referred to as a “personalised medicine”) approach may help the identification and management of stone-formers, it is worth considering its definition. NHS England defines personalised medicine as a “move away from a ‘one size fits all’ approach to the treatment and care of patients with a particular condition, to one which uses new approaches to better manage patients’ health and targets therapies to achieve the best outcomes in the management of a patient’s disease or predisposition to disease.” (https://www.england.nhs.uk/healthcare-science/personalisedmedicine/). Similarly, the NIH describe “precision medicine” as an approach for disease treatment and prevention that takes into account individual variability in genes, environment, and lifestyle for each person. Thus, this general view can be adopted to include the utilisation of both modern day investigations and more traditional approaches to understand more precisely and tailor the treatment of renal stone disease on an individual or patient by patient level. The combination of genetic and genomic information with other clinical and biochemical parameters will allow patterns of risk and disease susceptibility to be identified which should lead to earlier detection of illness and more effective interventions.

Furthermore, when considering genomic influence on disease, it is now imperative that we consider both the influences of the host genome, i.e. that of the patient as well as the host microbiome [22], the multitudes of microbes which colonise the body of the host. Within this microbiome, the number of bacterial cells is more than tenfold greater than the number of hosts cells [4]. Approaches to manipulate this distinctly separate but intimately related genome are being applied to kidney stone-formers and must be included in discussions relating to precision medicine approaches.

Traditional approaches to renal stone formation have typically considered environmental, geographic and dietary factors which might predispose to stone formation [33] and are often based on population studies. In addition, the heritability of renal stones has also been long established. Using twin studies, the estimated heritability of kidney stones was 56% [9], and this has been backed up by other studies assessing the familial pattern of stones [5, 17]. More recently, there has been the detailed search for rare monogenetic causes of renal stones using selected stone-forming populations [3, 6, 12]. Whilst rare monogenic forms of stone disease might initially appear a fine-print issue, to be reserved for the specialist, missed diagnoses of monogenetic causes of nephrolithiasis can lead to sub-optimal treatment, development of complications, including progression to end-stage renal disease, and failure to screen at risk family members [7].

Testing for known pathogenic mutations through high-throughput genetic analysis (such a gene panels and whole exome sequencing that modern next-generation sequencing techniques allow), is rapidly becoming an accessible diagnostic tool [3, 12]. The question remains whether this approach is worthwhile and fruitful in general calcium-forming stone populations. Indeed, in studies of hypercalciuric and hypocalciuric patients monogenic causes and rare alleles were not readily identified [32]. *CLCN5* variants were a plausible cause of idiopathic hypercalciuria but found to be rare [29]. Therefore, when considering performing genetic investigations on renal stone-formers, there is a balance of being able to discover a small yet significant number of monogenetic conditions (Table 1) versus the cost and resources that are required for such investigations. It could be argued that it is only when we obtain molecular precision in our diagnosis that we can expect to achieve accurate, personalised treatment for the patient. For the small number of patients with a monogenetic cause of their nephrolithiasis, a correct diagnosis may be crucial. Such monogenic disorders are likely to lead to increasing stone burdens and chronic kidney disease. Lack of genetic investigations may lead to delays in diagnosis, unscreened at risk family members [3, 12]. It is important to note that monogenic causes of stone, for example cystinuria, often produce recurrent stones, may present early in life and often lead to long-term serious complications, including end-stage renal disease [24].

**Table 1:** Monogenic causes of kidney stones, clinical features and suggested precision medicine treatments

Monogenetic condition	Inheritance pattern	Gene affected (MIM number)	Clinical features	Diagnostic implication and steps towards precision medicine
Infantile hypercalcaemia	Autosomal recessive; incomplete autosomal dominant	*CYP24A1* (126065)	Hypercalcaemia, elevated 1,25 (OH)_2_D, suppressed PTH, nephrolithiasis, nephrocalcinosis	Restrict excess vitamin D, avoid sun exposure and tanning beds Fluconazole treatment Rifampin treatment
Hypophosphataemic nephrolithiasis with osteoporosis	Autosomal dominant	*SLC34A1* (612286)	Renal phosphate wasting, nephrolithiasis	Familial screening Bone densitometry
Hypophosphataemic rickets with hypercalciuria	Autosomal recessive	*SLC34A3* (241530)	Hypophosphataemia Elevated 1,25 (OH)_2_D	Phosphate supplement Restrict Vitamin D
Autosomal-dominant hypocalcaemic hypercalciuria	Autosomal dominant	*CASR* (601198)	Basal ganglia calcifications, seizures, cramps, tetany nephrolithiasis	Familial screening, judicious calcium replacement, thiazide diuretics
Distal renal tubular acidosis	Autosomal dominant (and recessive)	*SLC4A1* (109270)	Acidosis, hypokalaemia. Growth impairment, nephrocalcinosis, nephrolithiasis. Haemolytic anaemia, spherocytosis/elliptocytosis	Bicarbonate and potassium replacement, familial screening. Monitor for CKD
Distal renal tubular acidosis	Autosomal recessive	*ATP6V1B1* (267300) *ATP6V0A4* (602722) FOXI1 (601093)	Acidosis, sensorineural hearing loss, rickets, osteomalacia	Bicarbonate and potassium replacement, familial screening Referral for audiometry Monitor for CKD
Cystinuria	Autosomal recessive and autosomal dominant with incomplete penetrance	*SLC3A1* (104614) *SLC7A9* (604144)	Elevated urine cysteine Recurrent stones—cysteine ± calcium stones, obstructive uropathy, pyelonephritis, chronic kidney disease	Family screening, urine alkalinisation, cysteine binding drugs, low animal protein diet, low sodium diet Stones may be resistant to shock wave lithotripsy Monitor for CKD
Xanthine oxidase deficiency (xanthinuria)	Autosomal recessive	XDH (278300)	Xanthine stones, low uric acid, myopathy	Avoid high purine foods Hydration Radiolucent stones
Primary hyperoxaluria type 1	Autosomal recessive	AGXT (604285)	Urinary oxalate raised, calcium oxalate stones, nephrocalcinosis, ESRD	Pyridoxine therapy (for certain mutations) Liver transplantation to prevent disease recurrence Monitor for CKD
Primary hyperoxaluria type 2	Autosomal recessive	GRHPR (260000)	Urinary oxalate raised, calcium oxalate stones, nephrocalcinosis, ESRD	Monitor for CKD
Primary hyperoxaluria type 3	Autosomal recessive	HOGA1 (613597)	Urinary oxalate raised, calcium oxalate stones	Limit hydroxyproline intake
Adenine phosphoribosyltransferase deficiency	Autosomal recessive	APRT (614723)	Excess urinary 2,8-dihydroxyadenine (DHA) 2,8-DHA kidney stones Intratubular and interstitial precipitation of DHA crystals leading to chronic kidney disease, ESRD	Allopurinol (xanthine dehydrogenase inhibitor) can prevent and dissolve kidney stones and improve kidney function Purine restriction Radiolucent stones Beware ESRD of unknown cause, may recur in renal transplant
Hypophosphataemic rickets with hypercalciuria	Autosomal recessive	SLC34A3 (241530)	Hypophosphataemia Elevated 1,25 (OH)_2_D	Phosphate supplement Restrict Vitamin D
Familial hypomagnesaemia with hypercalciuria and nephrocalcinosis	Autosomal recessive	CLDN16 (248250)	Myopia, hypercalciuria, nephrocalcinosis and nephrolithiasis ESRD may occur in second and third decade of life	Difficult to treat Thiazides may be tried Familial screening Monitor for CKD
Phosphoribosyl pyrophosphate synthase (PRPPS) hyperactivity	X-linked recessive	PRPS1 (311850)	Hyperuricaemia, uric acid stones	Allopurinol (xanthine dehydrogenase inhibitor) Purine restriction
Dent’s disease type 1 and 2 (Lowe syndrome)	X-linked recessive	CLCN5 (300008) OCRL (300535)	Low molecular weight proteinuria Hypercalciuria, Nephrolithiasis, nephrocalcinosis, ESRD	Thiazides may be tried Ophthalmologic screening Monitor for seizures Monitor for CKD

*CKD* chronic kidney disease, *ESRD* end-stage renal disease

## Spotting the genetic cases of renal stone disease: the diagnoses not to miss

The immediate priority for the physician treating the patient presenting with renal colic is to confirm the clinical diagnosis, alleviate pain and treat the presenting problem. The underlying causes and predisposing factors may not be at the forefront of considerations at this stage, and even with recurrent stone-formers, may be repeatedly neglected. Successfully delivering a personalised approach to stone-formers starts, in our opinion, with stone analysis, then proceeds to an individual assessment of lifestyle and biochemical parameters which may be predisposing to renal stone formation. Within medicine, it is often costly to ignore the opportunity for preventative intervention, both on an economic basis and in achieving good long-term patient outcomes. Accurately diagnosing the underlying cause of nephrolithiasis is crucial from a prognostic and therapeutic point of view [7]. *Common* causes of calcium oxalate nephrolithiasis must be delineated from *rare* causes, as the latter often carry a poorer prognosis with significant risk of progression to end-stage renal failure [7]. Stone-forming conditions such as cystinuria [24], primary hyperoxaluria [13] and adenine phosphoribosyltransferase (APRT) deficiency (producing 2,8-dihydroxyadenine stones) [26] are all also associated with risk of progression to end-stage renal disease, highlighting the crucial need for prompt diagnosis and preventative measures. The recently produced NICE guidance for renal and ureteric stones (https://www.nice.org.uk/guidance/indevelopment/gid-ng10033) advocates stone analysis in adult patients with stones.

As extreme as it may sound, it is possible for a missed diagnosis of a monogenic stone-forming condition such as primary hyperoxaluria or APRT deficiency to have devastating consequences. Missing a diagnosis of primary hyperoxaluria type 1 may lead to disease recurrence and oxalosis of a transplanted kidney [7], and the omission of the consideration of a curative liver transplantation. Undetected APRT deficiency can sometimes lead to ESRD and is also associated with recurrence of stone disease in a renal transplant, leading to loss of graft function if untreated [2].

Making a diagnosis in such cases ultimately relies on a combination of modalities, including stone analysis, urine biochemistry and genetic investigations. Whilst used as extreme examples they should also point to the fact that precision medicine approaches to stone-formers are not purely genetic but utilise the breadth of phenotypic information available.

## Promoting an individualised approach to stone-formers

The role of 24-h urine collections has been discussed in detail previously [15] and also in the accompanying discussion to this debate by Goldfarb. Whilst perceived to be a specialist and difficult to interpret test by the non-expert, a 24-h urine collection provides valuable and patient specific data allowing a precision medicine approach to be adopted [21]. An accurate assessment of urine volume over a 24-h time period is often very revealing, and distinct urinary biochemical profiles can lead to specific risk factors and diagnoses being made. It is our view that along with stone analysis, a 24-h urine collection, with all the caveats that it entails, pushes the patient towards a very much more individualised management pathway and based on its results also paves the way for genetic investigations to be considered.

A pragmatic view is that a genetic set of investigations is only likely to be requested if there is an unusual stone type, an unusual biochemical profile or a striking family history of renal stone disease or ESRD. Therefore, one could argue that whilst 24-h urines may be impractical and sometimes uninformative, it gives the personalised diagnostic pathway momentum which will drive consideration of genetic investigations. A 24-h urine collection or a spot urine evaluation for cystine (cystine/creatinine ratio) remains a valuable screening test in stone–forming patients, in order to identify cystinuria. Cystinuria patients may also be detected following stone analysis and microscopic examination of the urine [10]. Cystinuria accounts for 6–8% of stones in paediatric populations and around 1% of stones in adults [10] hence it is an important diagnosis to make, as the susceptibility to stones is lifelong. Traditionally, cystinuria patients and carriers have been attempted to be distinguished by cystine concentrations [11]. However, there are at least two genes implicated in cystinuria, *SLC3A1* labelled as type A and *SLC7A9*, labelled as type B. Inheritance patterns are mixed with autosomal recessive and dominant patterns with partial penetrance. It is possible to have digenic forms of the condition, for example type AB where two heterozygous variants (one from each gene) combine [24]. Indeed, sequencing of *SLC7A9* and *SLC3A1* allows a precise determination of genotype (and may reveal complex genotypes such as AAB and ABB, etc.) and allows at risk family members to be identified [24].

We have previously discussed in detail the importance of documenting a family history in the nephrology clinic [18]. In addition to this, age of onset of renal stone disease is particularly revealing. Paediatric patients presenting with early-onset of stone disease should arouse suspicion of a genetic cause and the clinician is led to look for these. However, when assessing the adult patient presenting with nephrolithiasis, clinicians may place less emphasis on family history, as they are not expecting an index presentation of a genetic disorder in this age group. However, a study using gene sequencing to analyse a cohort of 272 known stone-formers with no genetic diagnosis at the time, demonstrated that almost one-third of *SLC7A9* mutations (causing type B cystinuria) had an age of stone onset between 18 and 30 years, with a median age of 26 years [12]. Therefore, it is crucial to elicit a careful family history, and also look for any clues such as extra-renal manifestations (sensorineural hearing loss, neurological disorders) which may point towards a unifying underlying genetic disorder [7]. Other factors in the history, such as severity of disease or associated features such as nephrocalcinosis may also alert the clinician to search for a genetic cause [7]. Due to the varied clinical presentation and course of nephrolithiasis, absence of family history cannot be exclusively relied upon to exclude an underlying genetic cause; the clinician must remain vigilant to spot cases in which genetic testing may solve the diagnosis at an early stage and facilitate precise, individualised treatments.

Renal stones have a high frequency of recurrence. Biochemical profiles and stone-composition analysis can be helpful tools in establishing an etiological diagnosis for the patient. However, analysis of a single renal stone gives a single point-in-time reference and may be misleading. For example, some patients with cystinuria have been found to form calcium oxalate stones (as their presenting stone event) prior to the formation/identification of cysteine stones [25]. Therefore, analysis of a single stone, which on that occasion was a calcium-based stone, could lead to a false conclusion that this was a patient with common calcium nephrolithiasis, missing the underlying genetic cause and hence the opportunity to appropriately tailor their treatment.

Precision medicine thus relies on using targeted investigations at the right time for the right patient. Patients with an early age of renal stone onset are more likely to have an underlying monogenetic cause, and therefore genome-wide approaches such as whole exome sequencing (WES) may be an efficient method of determining the underlying diagnosis in patients presenting before the age of 25 [6]. The advantage of WES over gene-panel sequencing is that more genes than the already ‘known’ nephrolithiasis genes can be tested, allowing detection of novel candidate genes [3].

## Genetic cases of renal stone diseases: should you treat them differently?

Achieving a precise diagnosis of the underlying predisposition to nephrolithiasis is crucial when it comes to treatment options. Whilst generic treatment strategies such as maintaining adequate hydration might reduce stone burden in all stone-formers, this is a basic approach that neglects targeted therapies we know would be more successful in specific stone aetiologies.

For example, a low-salt diet may be generally helpful, but in cystinuria patients the priority should be to encourage a vegetarian or vegan diet (low in animal protein), aiming for an alkaline urine which reduces the likelihood of cystine stone formation. An alternative non-dietary method of achieving urinary alkalinisation is through potassium citrate supplementation. Patients can be educated about monitoring their urine pH, empowering them to lead on their individualised treatment plan. For patients with adenine phosphoribosyltransferase (APRT) deficiency, allopurinol is an effective treatment alongside generic fluid and dietary advice. These niche, inexpensive, simple treatments will only be initiated if the correct diagnosis has been made.

Reducing stone burden is crucial; whether you are trying to escape excruciating renal colic pain, or avoid surgical interventions or long-term renal damage, every stone is significant. Precise treatment, of a known monogenic cause (some examples are shown in Table 1), is preferable to empirical treatment which may well not be targeting the underlying problem. A detailed review of how these monogenic disorders provide opportunities for specific interventions has recently been published [23].

## Genetic investigations and gene discovery

With the growing ease and decreasing cost of genetic investigations it is notable that mutations in over 30 genes have been described in association with nephrolithiasis. Using next-generation sequencing approaches mutations were recently identified in *SLC26A1* in patients with calcium oxalate stones [8]. For *SLC26A1* mutations, it is not yet clear what the exact underlying pathophysiological mechanisms are, but this example serves as a reminder that aside from the known causes of nephronophthisis, others yet remain to be discovered. Indeed, recent descriptions of stone formation in patients with Pseudoxanthoma elasticum (secondary to mutations in *ABCC6*) points us towards new ways of modelling and understanding renal stone formation [16], and perhaps a rediscovery of previously postulated precision therapies, including pyrophosphate [19, 20].

## Inherited renal stone disease: it is all about being precise

Whilst renal stones can cause severe pain to the individual, on a population level only up to 10% of individuals are affected, and monogenetic cases of renal stone disease are even rarer [12]. However, making an early precise diagnosis of renal stone aetiology has a huge impact for the individual (and often other family members), allowing their treatment to be individualised and aiming to minimise any risk of progression to end-stage renal disease. Molecular genetic testing is becoming more accessible in a clinical rather than research context, and should be utilised where necessary to facilitate a precise diagnosis for the patient and their family. As clinicians we should aspire to maximise preventative interventions, appropriately using patient history, family history and clinical features such as extra-renal manifestations to identify cases which may be typical of monogenic disease. 24-h urine profiles when performed, will add to the diagnostic pathway and may reveal unexpected findings. Together these approaches should point the clinician towards genetic testing which may reveal vital diagnostic information. The priority should be to think ahead, trying to also target the disease process rather than react secondarily to stone burden, and tailoring the approach to the individual patient.

## The influence of the microbiome and stone disease

Microorganisms which colonise the human body are known collectively as the microbiome. In relatively recent studies, we are beginning to understand the importance of the role of the microbiome and how it communicates and influences the human body. Specifically the gut microbiome may influence the absorption and secretion of compounds implicated in renal stone formation. Distinct differences have been observed in the microbiome of renal stone-formers when compared to healthy controls [30, 31]. The identification of *Oxalobactor formigenes* [1], a bacteria which promotes the breakdown of oxalate in the gut, led to clinical trials using manipulation of the gut flora with dosing of *O. formigenes* [14]. These studies have led to more in-depth considerations of how an individual stone-former may be influenced by their own gut microbiome and directing therapies (such as pro-biotics) to manipulate this must be part of future precision medicine discussions for renal stone-formers.

## Conclusion

Renal stone disease is a huge burden on world health systems and any efforts directed at identifying an underlying and reversible cause should be undertaken. We advocate using a precision medicine approach to every stone-former which must include careful phenotyping, including stone analysis and 24-h urine collection, the results of which may prompt genetic testing. Empiric advice whilst welcome does not go far enough to target many of the known pathophysiological mechanisms of stone formation.
